# Exploring the Release of Toxic Oligomers from α-Synuclein Fibrils with Antibodies and STED Microscopy

**DOI:** 10.3390/life11050431

**Published:** 2021-05-11

**Authors:** Alessandra Bigi, Emilio Ermini, Serene W. Chen, Roberta Cascella, Cristina Cecchi

**Affiliations:** 1 Section of Biochemistry, Department of Experimental and Clinical Biomedical Sciences, University of Florence, 50134 Florence, Italy; alessandra.bigi@unifi.it (A.B.); emilio.ermini@stud.unifi.it (E.E.); 2 Centre for Misfolding Diseases, Department of Chemistry, University of Cambridge, Cambridge CB2 1EW, UK; serene_chen@bti.a-star.edu.sg

**Keywords:** synucleinopathies, protein aggregation, amyloid, toxic oligomers, Lewy bodies, PD, protein misfolding, neurodegeneration

## Abstract

α-Synuclein (αS) is an intrinsically disordered and highly dynamic protein involved in dopamine release at presynaptic terminals. The abnormal aggregation of αS as mature fibrils into intraneuronal inclusion bodies is directly linked to Parkinson’s disease. Increasing experimental evidence suggests that soluble oligomers formed early during the aggregation process are the most cytotoxic forms of αS. This study investigated the uptake by neuronal cells of pathologically relevant αS oligomers and fibrils exploiting a range of conformation-sensitive antibodies, and the super-resolution stimulated emission depletion (STED) microscopy. We found that prefibrillar oligomers promptly penetrate neuronal membranes, thus resulting in cell dysfunction. By contrast, fibril docking to the phospholipid bilayer is accompanied by αS conformational changes with a progressive release of A11-reactive oligomers, which can enter into the neurons and trigger cell impairment. Our data provide important evidence on the role of αS fibrils as a source of harmful oligomers, which resemble the intermediate conformers formed de novo during aggregation, underling the dynamic and reversible nature of protein aggregates responsible for α-synucleinopathies.

## 1. Introduction

The abnormal aggregation of αS, a 14 kDa intrinsically disordered protein implicated in neurotransmitter release at presynaptic terminals, is associated with the onset and progression of Parkinson’s disease (PD) and other debilitating neurodegenerative conditions collectively referred to as α-synucleinopathies [1]. Intraneuronal inclusions called Lewy bodies and neurites, primarily consisting of αS fibrils, are the major neuropathological hallmark of such diseases [2,3]. However, there is increasing evidence that metastable oligomeric assemblies, formed during the early phases of αS aggregation, play a crucial role in neuronal injury [4,5,6,7,8,9,10]. Despite their prominent role in the pathogenesis of α-synucleinopathies, the isolation and structural characterization of αS oligomers is extremely difficult because of their instability and transient nature. αS fibrils have also been demonstrated to be harmful, and their neurotoxicity has been associated with the disruption of plasma membrane integrity [11], with the perturbation of ionic homeostasis, as well as with the imbalance of cellular proteostasis systems [12]. Moreover, αS fibrils were reported to possess a high propagation propensity and to amplify the aggregation of endogenous αS by seeding [13,14,15,16,17].

The association of pathogenic αS species with lipid membranes is actually considered to be a primary event in the cascade of neuronal dysfunction in PD [8,9,10,11]. We have previously described the mechanisms of membrane perturbation and neuronal damage triggered by a range of well-characterized and stable αS aggregates, referred to as type-B* oligomers (OB*), short fibrils (SF), and long fibrils (LF), prepared under physiological conditions, according to developed protocols [5,7,10]. OB*, which possess a rudimentary cross-β structure and high solvent-exposed hydrophobicity [8,10], have been previously shown to be harmful to neuronal cells because of their ability to interact aberrantly with neurons and to rapidly penetrate cells [8,9,10]. Such oligomers can further aggregate and form fibrils, whose neurotoxicity has been associated with the release of oligomeric-like conformers, that are highly toxic to neuronal cells [5,7,10].

Taking advantage of a range of well-characterized conformation-sensitive antibodies selectively targeting αS conformers, and the super-resolution of STED microscopy, here we provide relevant evidence on the ability of SF to release small oligomers. These are structurally similar to OB* and penetrate human iPSC-derived dopaminergic neurons and primary rat cortical neurons. Moreover, we observed that the conformation-sensitive antibodies were able to bind to OB* and SF with a selective specificity, thus dramatically reducing their inherent neurotoxicity.

## 2. Materials and Methods

### 2.1. Generation of αS OB* and SF

The monomer of human αS (M) was expressed in *E. coli* BL21 cells (Agilent, Santa Clara, CA, USA) and then purified as previously reported [7]. OB* were generated by passing monomeric αS through a 0.22 μm cutoff filter and subsequently by incubating the protein at a concentration of 12 mg/mL in PBS buffer (pH 7.4) at 37 °C in stationary mode for 24 h, as reported previously [7,8]. SF samples were obtained by incubating monomeric αS at 70 μM in PBS buffer with a pH of 7.4 with 0.01% NaN_3_ at 37 °C under constant shaking for 4–6 days, and then by incubating 10 μM of these preformed fibrils with 100 μM monomeric αS for 13–15 h under quiescent conditions with subsequent sonication, as described previously [10]. The concentration of αS aggregates was estimated by measuring the absorbance at 275 nm using ε_275_ = 5600 M^−1^ cm^−1^ after the addition of 4 M guanidinium chloride.

### 2.2. Dot-Blot Assay of αS Conformers

To probe αS conformers, 0.7 μg of each sample (2 μL) was spotted onto a polyvinylidene fluoride (PVDF) membrane. The membranes were blocked for 30 min with 1.0% bovine serum albumin in TBS/TWEEN 0.1% and then incubated overnight at 4 °C with rabbit polyclonal anti-oligomer A11 antibodies (1:1000, Thermo Fisher Scientific, Waltham, MA, USA), rabbit polyclonal anti-amyloid fibrils OC antibodies (1:1000, Sigma-Aldrich, St. Louis, MO, USA), mouse monoclonal anti-aggregated αS 5G4 antibodies (1:1800, Sigma-Aldrich), or with mouse monoclonal anti-αS 211 antibodies (1:1800, Santa Cruz Biotechnology, Dallas, TX, USA). The immunolabelled blots were finally incubated with the proper secondary antibodies conjugated with horseradish peroxidase (Abcam, Cambridge, UK) and then detected by using a Pierce™ ECL Western Blotting Substrate (Thermo Fisher Scientific, Waltham, MA, USA) and ImageQuant™ TL software (GE Healthcare, Chicago, IL, USA).

### 2.3. Cell Cultures

Human SH-SY5Y neuroblastoma cells (A.T.C.C., Manassas, VA, USA) were cultured in Dulbecco’s Modified Eagle’s Medium (DMEM), F-12 Ham with 25 mM 4-(2-Hydroxyethyl)piperazine-1-ethanesulfonic acid (HEPES) and NaHCO_3_ (1:1) supplemented with 10% fetal bovine serum (FBS), 1.0 mM glutamine and 1.0% penicillin and streptomycin solution in a 5.0% CO_2_ humidified atmosphere at 37 °C and grown until 80% of confluence, as previously reported [9]. The cells were authenticated and tested to be free from mycoplasma contaminations.

Primary rat cortical neurons (Thermo Fisher Scientific) were plated and maintained in Neurobasal medium (Thermo Fisher Scientific) plus 0.5 mM GlutaMAX (Gibco, Thermo Fisher Scientific) and 2% (*v/v*) B-27 serum-free complement (Gibco, Thermo Fisher Scientific) at 37 °C in a 5.0% CO_2_ humidified atmosphere. Every 3–4 days, the medium was partially replaced and the experiments were performed 12–16 days after plating, as previously reported [18,19].

Human iPSC-derived dopaminergic neurons were obtained from iPSC-derived dopaminergic neuron progenitors (Axol Bioscience, Cambridge, UK). The cells were plated on 12-well plates containing glass coverslips coated with poly-D-lysine plus surebond-XF solution and led to maturation according to manufacturer’s instructions. The analyses were performed 14–18 days after plating, as previously described [10].

### 2.4. Analysis of the Interaction with and Internalization into Neuronal Cells of αS Conformers

SH-SY5Y cells seeded on glass coverslips were incubated for 60 min with αS conformers (0.3 μM) and then stained with 5.0 μg/mL Alexa Fluor 633-conjugated wheat germ agglutinin (Thermo Fisher Scientific), as previously reported [20]. Following a fixing step with 2% (*v/v*) paraformaldehyde, and a permeabilization phase with glycerol at 3.0% (*v/v*), αS was detected with rabbit polyclonal anti-oligomer A11 antibodies (1:250), rabbit polyclonal anti-amyloid fibrils OC (1:800), or with mouse monoclonal anti-αS 211 antibodies (1:250), then with Alexa-Fluor-488-conjugated anti-rabbit or mouse secondary antibodies (1:1000, Thermo Fisher Scientific). The detection of the fluorescence emission was obtained by double excitation at 633 nm and 488 nm through a TCS SP8 scanning confocal microscopy system (Leica Microsystems, Mannheim, Germany). A series of 1.0 μm thick optical sections (1024 × 1024 pixels) was taken through the cell depth for each sample using a Leica Plan Apo 63 × oil immersion objective and projected as a single composite image by superimposition. The confocal microscope was set and maintained constant at optimal detector gain and laser powers.

### 2.5. Analysis of the Mitochondrial Status with the MTT Assay

The mitochondrial functionality of the different αS species was evaluated in SH-SY5Y cells seeded in 96-well plates by the 3-(4,5-dimethylthiazol-2-yl)-2,5-diphenyltetrazolium bromide (MTT) assay. M, OB* and SF (0.3 μM concentration) were added to the culture medium of SH-SY5Y cells for 24 h. In a set of experiments, OB* and SF were added to the cell culture medium for 30 min and then rabbit polyclonal anti-oligomer A11 antibodies or rabbit polyclonal anti-amyloid fibrils OC antibodies, or mouse monoclonal anti-aggregated αS 5G4 antibodies, or mouse monoclonal anti-αS 211 antibodies were added (in a 1:2.5 molar ratio) for 24 h. The cell culture medium was then removed, cells were washed with PBS, and the MTT assay was performed as previously reported [10,18,19]. Cell viability was expressed as the percentage of MTT reduction in treated cells as compared to untreated cells, unless otherwise indicated.

### 2.6. Evaluation of Caspase-3 Activity

The activation of caspase-3, which is the primary effector caspase in the apoptotic pathway, was assessed in SH-SY5Y cells by confocal microscopy. αS species were added at 0.3 µM to the cell culture medium for 0, 1, 3, 5 and 24 h. In a set of experiments, SH-SY5Y cells were treated with OB* and SF and, after 30 min, the A11, OC, 5G4 and 211 antibodies were added to the cell culture medium for 24 h. After the incubation, the cell culture medium was removed and the FAM-FLICA Caspase 3/7 solution (Immunichemistry Technologies, LLC, Bloomington, MN, USA) was added as previously described [10]. The detection of the fluorescence emission was obtained by exciting the laser line at 488 nm through the confocal microscopy described in a previous paragraph. The different fluorescence intensities relative to activated caspase-3 were plotted versus the time elapsed after the addition of αS aggregates to the culture medium, and the resulting kinetic plots were fitted by means of a single exponential (1) or a sigmoidal (2) functions of the forms:

(1)  F(t)=F(eq)+Aexp(−kt)

(2) $$\;\;\;\;F\left(t\right)=F\;\left(eq\right)+\frac{F\left(0\right)-F\left(eq\right)}{1+{\left(\frac{kt}{A}\right)}^{B}}$$

where *F*(*t*) corresponds to the intracellular fluorescence at time *t* as a percentage of that observed in untreated cells, *F*(0) is the same fluorescence at 0 h, *F*(*eq*) is the same fluorescence at the apparent equilibrium (time ∞), *A* is the amplitude of the fluorescence change as a percentage of that observed in untreated cells, *k* is the apparent rate constant in s^−1^ and *B* is the slope of the sigmoidal function at time *t*.

### 2.7. Confocal Microscopy Analysis of αS Species In Vitro

OB* and SF (0.3 μM) were incubated in culture medium for 0 and 24 h at 37 °C in wells containing a glass coverslip in the absence of cells. The coverslips were then fixed with 2% (*v/v*) paraformaldehyde, blocked with 0.5% BSA to avoid non-specific antibody reactions, and incubated for 30 min at 37 °C with rabbit anti-oligomer A11 polyclonal antibodies (1:2500) or with mouse monoclonal anti-αS 211 antibodies (1:2000), then for 30 min with Alexa Fluor 514-conjugated anti-rabbit or anti-mouse secondary antibodies (1:2000, Thermo Fisher Scientific), as reported previously [10]. The detection of the fluorescence emission was obtained by exciting the laser line at 514 nm through the confocal microscope described previously.

### 2.8. Western Blotting of αS Species in Cellular Fractions

SH-SY5Y cells were treated with SF and OB*at 0.3 µM for 24 h. Membrane and cytoplasmic fractions were obtained as previously described with minor modifications [21]. Briefly, the cells were homogenized in PBS containing 9.0% sucrose with three freeze–thaw cycles, 5.0 s sonication on ice and centrifugation at 700× *g* for 10 min at 4 °C. The membrane fraction was pelleted by supernatant centrifugation at 110,000× *g* for 1 h at 4 °C and separated from the cytoplasmic one. The protein content in the fractions was measured by the method of Bradford [22]. Thirty µg of each fraction were run on 4–20% Mini-PROTEAN TGX precast gels (Bio-Rad, Hercules, CA, USA) and the separated proteins were blotted onto a Supported Nitrocellulose Membrane (Bio-Rad). The blotted membranes were then blocked in 1.0% (*w*/*v*) BSA in TBS-Tween (0.1% Tween 20 in 20 mM Tris–HCl buffer, pH 7.5, containing 100 mM NaCl) and then incubated with mouse monoclonal anti-αS 211 antibodies (1:250, Santa Cruz Biotechnology). After extensive washing, the membranes were incubated with peroxidase-conjugated anti-mouse secondary antibodies (Abcam) for 1 h and the immunolabelled bands were detected using a Pierce™ ECL Western Blotting Substrate (Thermo Fisher Scientific) and ImageQuant™ TL software (GE Healthcare).

### 2.9. STED Microscopy Analysis of αS Species in Neurons

Primary rat cortical neurons and human iPSC-derived dopaminergic neurons were treated with OB* and SF at 0.3 µM for 24 h. After treatment, cortical neurons were stained with 0.01 mg/mL wheat germ agglutinin, Tetramethylrhodamine Conjugate (TMR, Thermo Fisher Scientific) and αS was detected with mouse monoclonal anti-αS 211 antibodies (1:125) and Alexa Fluor 514-goat anti-mouse secondary antibodies (1:500) as previously reported [10]. Dopaminergic neurons were stained with mouse anti-MAP-2 antibodies (1:400, Abcam) and rabbit polyclonal anti-oligomer A11 antibodies (1:250) and then with Alexa-Fluor-568-conjugated anti-rabbit secondary antibodies (1:500, Thermo Fisher Scientific) and Alexa-Fluor-514-conjugated anti-mouse secondary antibodies (1:500, Thermo Fisher Scientific). Fluoromount-G™ (Thermo Fisher Scientific) was used as mounting medium. Then, the STED images (i.e., z-stacks acquired along three directions: x, y, and z axes) were acquired by using an SP8 STED 3X confocal microscopy (Leica Microsystems). The detection of the fluorescence emission was obtained after double excitation at 550 nm or 568 nm, and 514 nm through a white light laser (WLL). STED xyz images were acquired in bidirectional mode. The emitted fluorescence of TMR, Alexa Fluor 568 and Alexa Fluor 514 were collected from 564 to 599 nm, from 575 to 595 nm, and from 532 to 551 nm, respectively. Frame sequential acquisition was applied to avoid fluorescence overlap. Gated pulsed-STED was applied to Alexa-Fluor 514 fluorophore, with a gating between 0.3 to 6 ns, to avoid the collection of reflection and autofluorescence. A Leica HC PL APO CS2 100×/1.40 oil STED White objective was used. Images were collected at 0.1 μm intervals with a Z stack of approximately 5 μm and deconvolved with Huygens Professional software (Scientific Volume Imaging B.V., Hilversum, The Netherlands; version 18.04), as previously reported [10].

### 2.10. Statistical Analysis

All data were presented as a means ± standard error of mean (S.E.M). Comparisons between the different groups were performed by the Student *t*-test or by ANOVA followed by Bonferroni’s post-comparison test by using GraphPad Prism 7.0 software (San Diego, CA, USA).

## 3. Results

### 3.1. αS Oligomers Permeabilize Neuronal Membrane Triggering Cell Dysfunction

Due to the transient nature and high structural heterogeneity of intermediates formed during αS aggregation, in this study we employed a set of conformation-sensitive antibodies to target the different αS conformers by immunoblot analysis. Specifically, we assessed the degree of specificity of A11, OC and 5G4 antibodies for the well-characterized and stable αS M, OB* and SF, prepared under physiological conditions according to developed protocols [5,7,10]. The A11 antibodies have been previously reported to recognize a structural epitope present in oligomeric assemblies of different proteins and peptides, which is absent in monomers and fibrils [23]. The OC antibodies are specific for amyloid fibrillar species without labelling monomers and oligomers [24], whereas the 5G4 antibodies were raised against every αS aggregated species [25,26].

**Figure 1:**
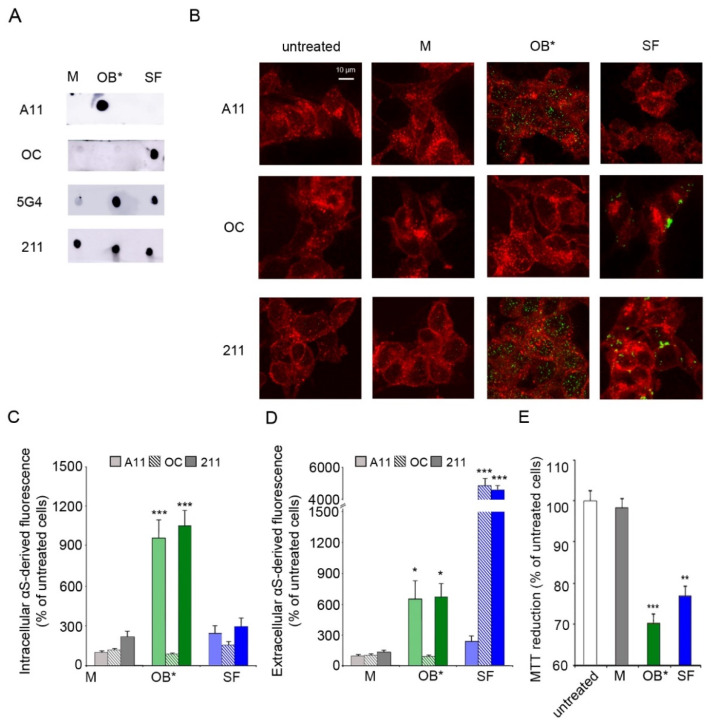
(**A**) Dot-blot analysis of αS conformers labelled with conformation-sensitive antibodies A11, OC, and 5G4, and conformation-insensitive 211 antibodies specific for human αS. (**B**) Representative confocal images of SH-SY5Y cells exposed to αS species at 0.3 μM for 1 h. Red fluorescence shows the cell membranes revealed with wheat germ agglutinin. Green fluorescence indicates the αS species labelled with the indicated antibodies. (**C**,**D**) Histograms reporting the semi-quantitative analysis of intracellular (**C**) and extracellular (**D**) αS fluorescence expressed as the percentage observed in untreated cells, taken as 100%. (**E**) MTT reduction in SH-SY5Y cells exposed to αS species at 0.3 µM for 24 h. Error bars indicate S.E.M. The statistical analysis was made by one-way ANOVA followed by Bonferroni’s multiple comparison test relative to untreated cells (* *p*  <  0.05, ** *p*  <  0.01, *** *p*  <  0.001).

The dot-blot analysis showed that the A11 and OC antibodies can selectively detect αS oligomers and fibrils, respectively (Figure 1A). In contrast, the 5G4 antibodies recognized both αS aggregates, with a very low cross-reaction with the monomeric protein. The equal loading of each αS conformer was assessed by the monoclonal conformation-insensitive anti-αS 211 antibodies, which are specific for residues 121–125 at the C-terminal domain of human αS (Figure 1A).

We then evaluated the ability of the conformation-sensitive antibodies to detect αS conformers interacting with the plasma membrane (extracellular αS) and being internalized into the cytosol of fixed human SH-SY5Y neuroblastoma cells (intracellular αS), which were previously incubated for 1 h with culture medium containing αS species at a concentration of 0.3 μM. The plasma membranes (red channel) were counterstained with wheat germ agglutinin, and αS species (green channel) with the aforementioned antibodies (A11, OC and 211), and subsequently analyzed by confocal scanning microscopy (Figure 1B). A very low green fluorescent signal was observed both intracellularly and extracellularly with A11, OC and 211 antibodies in cells exposed to αS M (Figure 1B,C). Notably, the A11 antibodies showed a significant increase in intracellular αS-derived fluorescence (by 956 ± 130%) in neuronal cells exposed to OB* relative to untreated cells, taken as 100%. On the contrary, a low intracellular αS A11-reactivity was shown in cells treated with SF (by 241 ± 51%) (Figure 1B,C). In addition, the OC antibodies selectively targeted SF at the surface of the neuronal membranes (by 4891 ± 320%), without any positive reactivity in cells exposed to OB* (Figure 1B,D). In contrast, the 211 antibodies were able to recognize both OB* and SF interacting with the surface of the plasma membrane (by 669 ± 128% and 4641 ± 227%, respectively) (Figure 1B,D) and being internalized by neuronal cells (by 1055 ± 106% and 293 ± 60%, respectively) (Figure 1B,C). These results are in good agreement with previously reported data [8,10], suggesting that OB* have a much higher propensity to permeabilize the neuronal membrane with respect to SF, which remained largely localized at the membrane surface.

We then evaluated whether the permeabilizing ability of αS species was related to their capacities to induce mitochondrial dysfunction. The ability of SH-SY5Y cells to reduce the 3-(4,5-dimethylthiazol-2-yl)-2,5-diphenyltetrazolium bromide (MTT) significantly decreased (by 28 ± 2%), following a 24 h treatment with 0.3 µM OB*, according to our previous results [27]. SF, which have been found to be largely bound to the outer leaflet of the neuronal membranes at 1 h of exposure, were able to significantly decrease the MTT reduction (by 23 ± 2%) at longer time of exposure, whereas M appeared to be harmless (Figure 1E).

**Figure 2:**
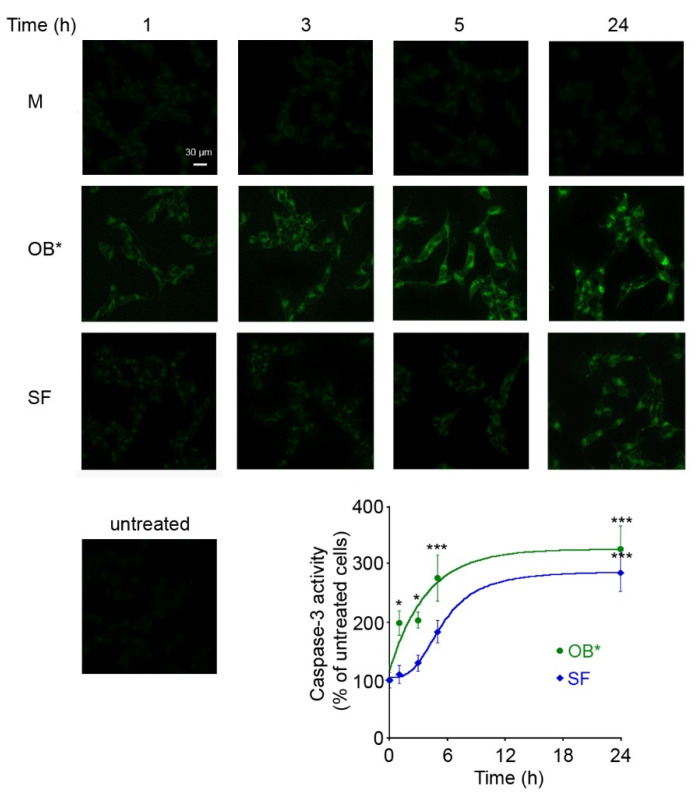
Representative confocal images of caspase-3 activity in SH-SY5Y cells treated for different lengths of time (1, 3, 5 and 24 h) with M, OB* and SF at 0.3 µM. Untreated cells are also shown. The fluorescence signals are expressed as the percentage of the values for untreated cells, taken as 100%. The kinetic plots report the caspase-3 derived fluorescence versus time following αS addition to the cell medium. The continuous lines through the data represent the best fits to a single-exponential function for OB* (green line), and a sigmoidal function for SF (blue line). Error bars indicate S.E.M. The statistical analysis was made by one-way ANOVA followed by Bonferroni’s multiple comparison test relative to untreated cells (* *p* < 0.05, *** *p* < 0.001).

To clarify whether the toxicity of αS conformers depends on the time of exposure to neuronal cells, we monitored over time the ability of OB* and SF to cause the activation of caspase-3, the most common marker of apoptosis [28]. OB* were more rapid, with respect to SF, in inducing caspase-3 activation, with a half-maximal time of apoptotic activation occurring after 3 h of treatment with OB* and after 6 h with SF (Figure 2).

Indeed, the maximal toxicity of SF was observed following 24 h of cell exposure, when the fluorescent signals were expressed as the percentage of the values for untreated cells, taken as 100%. This evidence suggests that OB* cause an immediate cell dysfunction, whereas fibrils induce gradual deleterious effects on neuronal cells.

### 3.2. Conformation-Sensitive Antibodies Attenuate the Toxicity of αS Aggregates

**Figure 3:**
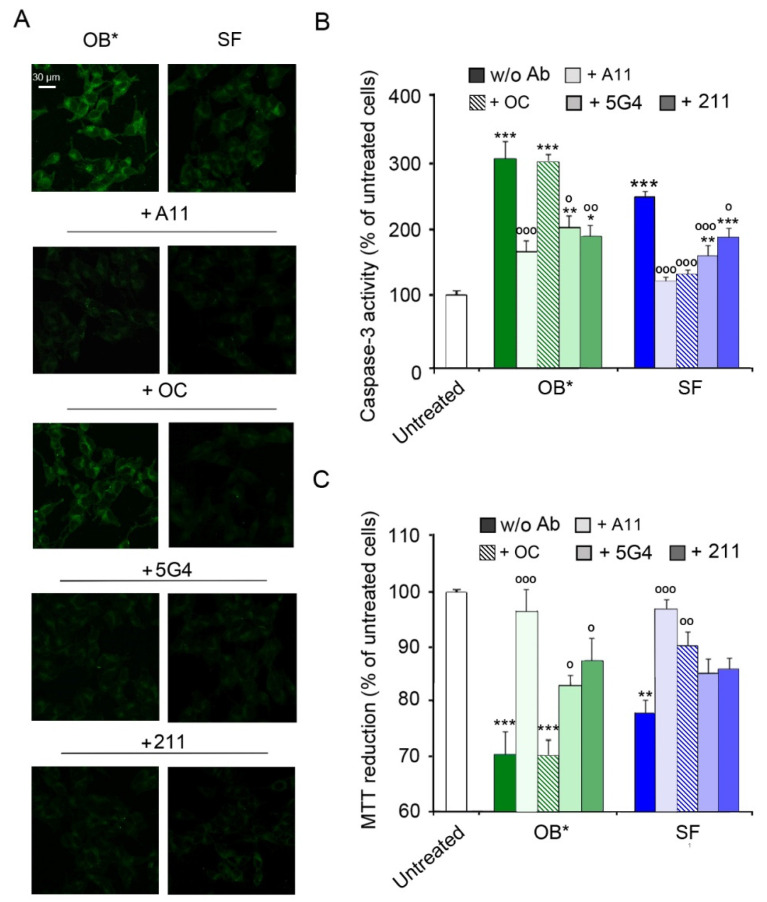
(**A**) Representative confocal images showing caspase-3 activity in SH-SY5Y cells treated with αS aggregates at 0.3 µM and A11, OC, 5G4 and 211 antibodies (in a 1:2.5 molar ratio) for 24 h. (**B**) The histogram reports a semi-quantitative analysis of caspase-3 activity expressed as the percentage of untreated cells represented in Figure 2, taken as 100%. (**C**) MTT reduction in SH-SY5Y cells treated with αS aggregates (0.3 µM) and with A11, OC, 5G4 and 211 antibodies (in a 1:2.5 molar ratio) for 24 h. Error bars indicate S.E.M. The statistical analysis was made by one-way ANOVA followed by Bonferroni’s multiple comparison test relative to untreated cells (* *p* < 0.05, ** *p* < 0.01, *** *p* < 0.001), or to cells treated with the same αS species without antibodies (° *p* < 0.05, °° *p* < 0.01, and °°° *p* < 0.001).

We then exploited the conformation-sensitive antibodies in competition experiments to investigate the mechanisms by which SF cause a delayed neuronal dysfunction. Specifically, we compared the activation of the apoptotic pathway in SH-SY5Y cells exposed to αS conformers and A11, OC, 5G4 and 211 antibodies (in a αS:Ab molar ratio of 1:2.5) for 24 h. Similarly to the results of the time course analysis, OB* and, to a lesser extent, SF, induced a significant caspase-3 activation (by 305 ± 25% and 251 ± 8%, respectively, compared to untreated cells) in the absence of antibodies (Figure 3A,B). Notably, the addition of A11 antibodies significantly prevented apoptosis activation induced by both OB* and SF (by 132 ± 40% and 124 ± 13%, respectively). In contrast, the OC antibodies markedly rescued the caspase-3 activation evoked by SF (by 114 ± 14%), without affecting OB* cytotoxicity (Figure 3A,B). Accordingly, a protective effect of 5G4 antibodies against apoptotic death triggered by OB* and SF (by 98 ± 41% and 88 ± 20%, respectively) was also observed. The 211 antibodies significantly reduced the activation of the apoptotic pathway induced by OB* (by 111 ± 39%), with a minor inhibition of caspase-3 activation induced by SF (by 59 ± 18%; Figure 3A,B).

A consistent trend was obtained with the analysis of metabolic impairment by the MTT assay (Figure 3C). Indeed, the A11 antibodies significantly prevented the cytotoxicity induced by both OB* (by 27 ± 8%) and SF (by 22 ± 6%). In contrast, the OC antibodies significantly prevented the neurotoxicity of SF (by 14 ± 4%), but not that of OB* (Figure 3C). The 5G4 antibodies significantly inhibited the OB*-induced mitochondrial dysfunction (by 14 ± 5%), with only a minor and not significant effect against SF (Figure 3C). Finally, the presence of the conformation-insensitive 211 antibodies significantly rescued the metabolic impairment induced by OB* (by 18 ± 7%), with a slight but not significant effect against the fibrillar species (Figure 3C). Taken together, these results indicate that A11 antibodies can significantly rescue the neurotoxicity of both oligomeric and fibrillar αS conformers, whereas the OC antibodies can inhibit only the fibril-induced cellular dysfunction. These data also suggest that A11 antibodies can bind OB*-like oligomers released by SF, thus preventing apoptosis in neuronal cells surrounded by fibrils.

### 3.3. αS Fibrils Progressively Release Oligomers In Vitro and In Vivo

**Figure 4:**
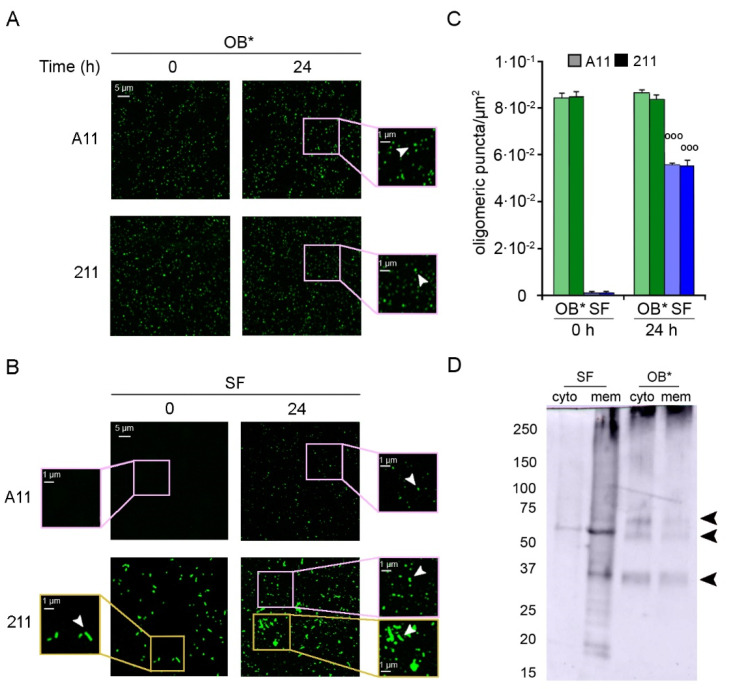
(**A**,**B**) Representative confocal images showing OB* (**A**) and SF (**B**) incubated in a glass coverslip at 0.3 μM in SH-SY5Y culture medium without cells for 0 and 24 h at 37 °C. The green-fluorescent signals arise from the staining with the A11 and 211 antibodies, respectively. The pink and yellow boxed areas show higher magnifications of the αS species. (**C**) Semi-quantitative analysis of the oligomeric αS-derived green fluorescent signal showed in panels A and B, and expressed as number of puncta per µm^2^. Error bars indicate S.E.M. The statistical analysis was made by Student *t*-test relative to samples of SF at time 0 h (°°° *p* < 0.001). (**D**) Western Blotting of the cytosolic (cyto) and membrane (mem) fractions purified from SH-SY5Y cells treated for 24 h with SF or OB* at 0.3 μM. αS species were then probed with conformation-insensitive anti-αS 211 antibodies (Original Western Blot Figure see Figure S1).

To analyze the possible leakage of A11-reactive oligomers from fibrils, we incubated αS aggregates in the culture medium in the absence of cells on a glass coverslip for 24 h at 37 °C. Then, the αS species were labelled with the conformation-sensitive A11 antibodies, as well as with the conformation-insensitive 211 antibodies. The emitted fluorescence was detected at 514 nm excitation line by the confocal scanning microscopy system (Figure 4).

Small dots of αS-derived oligomers were counted using ImageJ software after setting constant thresholds for every image, and excluding fibrillar species from the particle counts. Both antibodies revealed the presence of a homogeneous population of small and globular oligomers in freshly prepared OB* samples (at time 0 h) and in OB* samples incubated for 24 h at 37 °C (Figure 4A, arrowheads in the right image magnifications), with the amount of puncta remaining constant over time (~8.4 × 10^−2^ puncta/µm^2^) (Figure 4C). On the contrary, the A11-reactive aggregates were substantially absent in freshly prepared SF samples (at time 0 h) (Figure 4B, left image magnification), and appeared after 24 h of incubation at 37 °C (Figure 4B, arrowhead in the right image magnification). Specifically, the amount of oligomeric puncta significantly increased at 24 h (~5.5 × 10^−2^ puncta/µm^2^) (Figure 4C), suggesting a release of oligomeric conformers by mature fibrils over time. Accordingly, after a 24 h incubation of SF sample in cell culture medium, the conformation-insensitive 211 antibodies detected the appearance of a population of small green fluorescent dots (Figure 4B, arrowhead in the right upper image magnification), likely oligomeric puncta (Figure 4C), together with the presence of fibrillar species (Figure 4B, arrowhead in the right lower image magnification).

We then explored whether the release of oligomers from αS fibrils was also evident in different subcellular compartments of neuronal cells. After a 24 h treatment with OB* or SF, SH-SY5Y cells were collected and fractionated to isolate membrane and cytosolic fractions. The cellular fractions were then analyzed by using the mouse monoclonal 211 antibody and western blotting assay (Figure 4D). The membrane fraction of cells treated with SF showed a smear with a continuous of bands at the top of the lane and two intense bands at ~35 KDa and ~60 KDa (indicated by arrowheads) and some weaker bands at lower molecular weight. In contrast, the cytosolic sample contained only a milder band at ~60 kDa (indicated by arrowhead), suggesting that a low amount of small-size αS was internalized in SF-treated cells (Figure 4D). Both fractions of cells treated with OB* showed a similar pattern with a smear at the top of the gel and three clearer bands at ~35 KDa, ~60 KDa and ~70 kDa (indicated by arrowheads). However, the cytosolic fraction showed higher amounts of internalized αS than the membrane one (Figure 4D). These results indicate a gradual leakage of small species from fibrils bound to the plasma membranes, which can insert into the lipid bilayer and reach the intracellular compartment of neuronal cells.

### 3.4. The Penetration of αS Oligomers into Cortical and Dopaminergic Neurons by STED Microscopy

**Figure 5:**
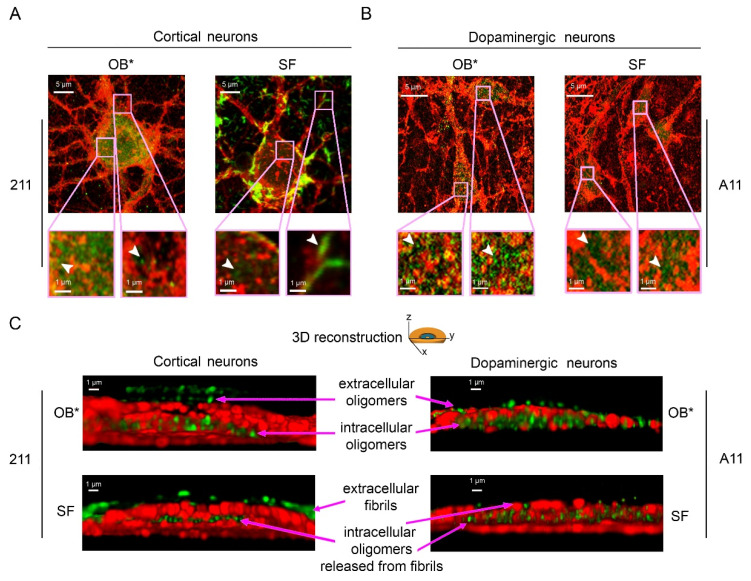
Representative STED images of primary rat cortical neurons (**A**) and human iPSC-derived dopaminergic neurons (**B**) exposed to OB* and SF at 0.3 µM for 24 h. Red and green fluorescence indicates the cell membranes and the αS species labelled with wheat germ agglutinin and the conformation-insensitive human-specific anti-αS 211 antibodies, respectively, in (**A**), or by MAP-2 and the anti-oligomer A11 antibodies, respectively, in (**B**). The pink boxed areas show higher magnifications of the αS species. (**C**) 3D reconstructions of the z-stack analysis of the cells shown in panels A and B. Neurons were virtually dissected on the zy plane to show more clearly the extracellular (top) and intracellular (middle) αS species labelled with 211 (left images) and A11 antibodies (right images).

To better characterize the subcellular structures of αS species bound to the plasma membrane and internalized into the neurons, we took advantage of the nanometric precision of the super-resolution STED microscopy. Primary rat cortical neurons treated with OB* for 24 h showed a large number of green-fluorescent puncta, labelled with the conformation-insensitive anti-αS 211 antibodies, both outside and inside the cells (Figure 5). These assemblies were small and globular in shape (Figure 5A, arrowheads in the left and right image magnifications). According to the above reported evidence, neurons exposed to SF for 24 h showed αS fibrillar species bound to the external side of the plasma membrane (Figure 5A, arrowhead in the right image magnification). The same images also show a range of small and globular intracellular αS species, possessing a morphology that was very similar to that of OB* (Figure 5A, arrowhead in the left image magnification). We also probed the nature of the αS species able to penetrate into human iPSC-derived dopaminergic neurons, a relevant PD model, by using the conformation-sensitive A11 antibodies (Figure 5B). STED images revealed a conspicuous A11-positive signal in neurons treated with both OB* and SF for 24 h (Figure 5B, arrowheads in the image magnifications), strongly indicating the presence of αS oligomeric conformers similar to OB* inside dopaminergic neurons treated with SF.

To gain mechanistic details into the binding of αS aggregates to the neuronal membranes and their internalization into the cells, we performed a z-stack analysis to obtain the 3D reconstruction of the cortical and dopaminergic neurons treated with OB* and SF (Figure 5C).

The mouse monoclonal 211 antibodies revealed the presence of oligomeric assemblies bound to the outer leaflet of the plasma membrane and internalized into the cytosol in cortical neurons treated with OB* (Figure 5C, upper left image). By contrast, cortical neurons exposed to SF presented elongated aggregates attached to the extracellular side of the membrane, along with intracellular species showing a globular morphology similar to that of the aggregates observed inside neurons exposed to OB* (Figure 5C, lower left image). In addition, by using the conformation-sensitive and oligomer-specific A11 antibody, we observed the presence of oligomeric assemblies inserted into the lipid bilayer and internalized into the cytosol of dopaminergic neurons treated with SF, as well as with OB* (Figure 5C, lower and upper right images, respectively). Overall, these data provide relevant evidence that the αS fibrils release small oligomers bearing membrane-disrupting properties, which are crucial to enter deeply into the bilayer interior and inside the affected neurons.

## 4. Discussion

αS prefibrillar oligomers are considered to play a prominent role in the massive loss of dopaminergic neurons responsible for the onset and progression of PD [4,5,29,30]. Despite the increasing relevance given to αS oligomeric assemblies for their ability to induce neuronal dyshomeostasis, many findings support the toxic capacity of mature fibrils [31]; indeed, the exposure of neuronal cells to such assemblies induces the aggregation of the endogenous αS and the formation of inclusions that are morphologically and biochemically similar to those found in the brain of PD patients [13,14,15,16,17]. When different αS aggregates were injected into the mouse striatum to determine their ability to induce PD-related phenotypes, β-sheet rich oligomers, referred to as OB*, were found to produce a small but significant loss of dopaminergic neurons in the *substantia nigra* (SN). The injection of short β-sheet fibrils (SF), however, produced the most robust PD-like phenotypes, including the reduction of striatal dopamine terminals, the massive loss of dopaminergic neurons in SN, and evident motor-behavior defects [17]. Accordingly, the aggregation of αS into insoluble fibers accumulated in Lewy bodies has been associated with the degeneration of dopaminergic neurons in SN [13,32,33,34].

Recent evidence obtained in relevant neuronal models indicates that OB* rapidly induce membrane perturbation, associated with calcium dyshomeostasis and production of reactive oxygen species, whereas SF are toxic after prolonged times of binding to the membrane [7,8,9,10,35,36,37,38]. The mechanism of interaction between αS oligomers/fibrils and lipid membranes has been extensively investigated, as it is a crucial event in PD pathophysiology [30,31,39,40]. Relevant studies indicated that OB* expose the disordered lipophilic N-terminus of monomer subunits, which anchors oligomers into the membrane, thus facilitating the insertion of the rigid oligomer core deeply into the hydrophobic interior of the membrane [8,9,30]. On the contrary, the burying of lipophilic N-terminus of monomers within non-toxic αS oligomers referred to as OA*, precludes their insertion in the interior of the bilayer [8,30]. However, this issue needs further investigation, both in terms of formation of mature fibrils from oligomeric conformers and dissolution of fibrils into soluble and harmful oligomers upon the interaction with phospholipid bilayers.

In this work, we took advantage of a range of well-characterized conformation-sensitive antibodies, able to selectively target the specific conformations of misfolded oligomers and fibrils, and the super-resolution STED microscopy, to give further insights to our previously reported evidence on the capability of αS fibrils to release oligomeric species, both in vitro and on the neuronal bilayer [10]. Here, we found that the ability of OB* to cause neuronal toxicity is strongly associated with their rapid and massive adsorption onto the plasma membrane and internalization into the cytosol, occurring already after 1 h upon their addition to the cell culture medium.

In contrast, the preformed SF remain mainly localized at the outer leaflet of the phospholipid membrane after prolonged exposure to cortical and dopaminergic neurons, as revealed at high resolution by STED microscopy. The presence of αS oligomeric species similar to OB* inside the cells upon 24 h of treatment with SF strongly suggests that the cytotoxicity of SF cannot be associated with their superficial interaction with neuronal membranes, but rather to their ability to dissolve in membrane-disrupting oligomers, which can penetrate neurons. Accordingly, western blotting analysis and biological read-outs collectively show that the toxic effects of the fibrils correlate with the ability of the released oligomers to be internalized into the cytoplasm, causing a massive caspase-3 activation and mitochondrial dysfunction. In particular, our results indicate that αS species with a β-sheet conformation and solvent-exposed hydrophobic surfaces may affect neuronal viability, although at different times of exposure and with distinct levels of toxicity. Taking advantage of STED microscopy and the conformation-sensitive A11 antibody, which are specific for ‘soluble prefibrillar oligomers’ [23], we found that the species able to penetrate dopaminergic neurons were A11-positive in all cases, not only when the cells were facing OB*, but also SF, that are A11-negative per se.

Furthermore, in a cell-free condition, SF were found to release A11-positive species over time. These results agree with the previously described capability to disaggregate of αS fibers incubated in vitro under near-physiological conditions (pH 7.4, 37 °C) in monomer-free buffer, thus leading to the release of small oligomeric assemblies [5]. Moreover, the ability of αS and also β_2_-microglobulin fibrils to fragment into toxic oligomeric conformers has been observed in vitro upon mild pH acidification [41,42]. Low pH is also characteristic of cellular organelles like lysosomes, a condition that could enhance the release of small oligomers from intraneuronal deposits, possibly able to spread to nearby cells [41]. Importantly, soluble, highly stable, and potentially toxic annular αS oligomers were previously reported to be released upon a mild detergent treatment from glial cytoplasmic inclusions obtained from brain tissues of patients affected by multiple system atrophy [43]. Accordingly, it was also shown that amyloid beta (Aβ) fibrils accumulated in senile plaques in the brain of Alzheimer’s disease (AD) patients, can resolubilize into A11-positive oligomers sharing biophysical and toxic properties with those formed in the very early phases of Aβ aggregation [44]. This evidence is also consistent with the observation of the leakage of Aβ oligomers from fibrils in the mouse brain [45]. In particular, the toxicity observed in a mouse model overexpressing the Aβ peptide was proportional to the quantity of oligomers released from amyloid plaques. Furthermore, those plaques were neighbored by oligomeric Aβ aberrantly interacting with synapses and contributing to their loss [45].

Taken together, our results support the claim that fibrils formed by different proteins release oligomers, that can, in turn, contribute to the toxicity associated with fibril spreading, and reinforce the idea that protein aggregates responsible for misfolding diseases such as α-synucleinopathies are highly dynamic and can interconvert each other on lipid membrane surfaces under specific microenvironmental conditions. Thus, both αS oligomers and fibrils are involved in neurodegeneration, with mature fibers acting as a source of soluble oligomers, which are the toxic moiety promptly affecting neuronal cells.

Our experimental data also suggest that antibodies against αS aggregates, directly interacting with such species, or preventing the release of toxic oligomers from mature fibrils, might be suitable tools for the development of new therapeutic strategies against PD and α-synucleinopathies in general. Accordingly, conformation-specific antibodies, recently developed against αS oligomers and fibrils, showed promising results both in cellular and animal models [46,47], suggesting their potential assessment in clinical trials [48,49,50]. Such antibodies offer the indisputable advantage of specifically targeting pathogenic αS aggregates without affecting the physiological functions of αS monomer.

## 5. Conclusions

Our evidence reinforces the idea that αS fibrillar aggregates can represent a fount of harmful soluble oligomers, bearing membrane-disrupting properties. Thus, the conformational characterization of the pathologically relevant species using super-resolution techniques is of fundamental importance to shed light on the molecular basis of synucleinopathies and provide innovative insight into rational drug development.

## Data Availability

All data supporting the findings of this study are available upon reasonable request to the corresponding authors.

## Supplementary Materials

The following are available online at https://www.mdpi.com/article/10.3390/life11050431/s1, Figure S1: Western Blotting of the cytosolic (cyto) and membrane (mem) fractions purified from SH-SY5Y cells treated for 24 h with SF or OB* at 0.3 μM. αS species were then probed with conformation-insensitive anti-αS 211 antibodies.
