# Supplementary material for: Exploring the Release of Toxic Oligomers from α-Synuclein Fibrils with Antibodies and STED Microscopy
Source: Life (Basel). 2021 May 11;11(5):431. doi: 10.3390/life11050431 (PMC8150853; doi:10.3390/life11050431)
Supplement: Supplementary file 1 [file life-11-00431-s001.zip › life-1187692-supplementary.pdf]

life

MDPI

Article

Exploring the Release of Toxic Oligomers from α-Synuclein Fibrils with Antibodies and STED Microscopy

Alessandra Bigi [1], Emilio Ermini [1], Serene W. Chen [2], Roberta Cascella [1,*] and Cristina Cecchi [1,*]

[1] Section of Biochemistry, Department of Experimental and Clinical Biomedical Sciences, University of Florence, 50134 Florence, Italy; alessandra.bigi@unifi.it (A.B.); emilio.ermini@stud.unifi.it (E.E.)
[2] Centre for Misfolding Diseases, Department of Chemistry, University of Cambridge, Cambridge CB2 1EW, UK; serene_chen@bti.a-star.edu.sg
* Correspondence: roberta.cascella@unifi.it (R.C.); cristina.cecchi@unifi.it (C.C.); Tel.: +39-055-2751223 (R.C.); +39-055-2751222 (C.C.)

Abstract: α-Synuclein (αS) is an intrinsically disordered and highly dynamic protein involved in dopamine release at presynaptic terminals. The abnormal aggregation of αS as mature fibrils into intraneuronal inclusion bodies is directly linked to Parkinson's disease. Increasing experimental evidence suggests that soluble oligomers formed early during the aggregation process are the most cytotoxic forms of αS. This study investigated the uptake by neuronal cells of pathologically relevant αS oligomers and fibrils exploiting a range of conformation-sensitive antibodies, and the super-resolution stimulated emission depletion (STED) microscopy. We found that prefibrillar oligomers promptly penetrate neuronal membranes, thus resulting in cell dysfunction. By contrast, fibril docking to the phospholipid bilayer is accompanied by αS conformational changes with a progressive release of A11-reactive oligomers, which can enter into the neurons and trigger cell impairment. Our data provide important evidence on the role of αS fibrils as a source of harmful oligomers, which resemble the intermediate conformers formed de novo during aggregation, underling the dynamic and reversible nature of protein aggregates responsible for α-synucleinopathies.

Keywords: synucleinopathies; protein aggregation; amyloid; toxic oligomers; Lewy bodies; PD; protein misfolding; neurodegeneration

Citation: Bigi, A.; Ermini, E.; Chen, S.W.; Cascella, R.; Cecchi, C. Exploring the Release of Toxic Oligomers from α-Synuclein Fibrils with Antibodies and STED Microscopy. *Life* **2021**, *11*, 431. https://doi.org/10.3390/life11050431

Academic Editors: Giuliana Fusco and Stefano Gianni

Received: 16 April 2021
Accepted: 4 May 2021
Published: 11 May 2021

Publisher's Note: MDPI stays neutral with regard to jurisdictional claims in published maps and institutional affiliations.

Copyright: © 2021 by the authors. Licensee MDPI, Basel, Switzerland. This article is an open access article distributed under the terms and conditions of the Creative Commons Attribution (CC BY) license (http://creativecommons.org/licenses/by/4.0/).

Supplementary Materials:

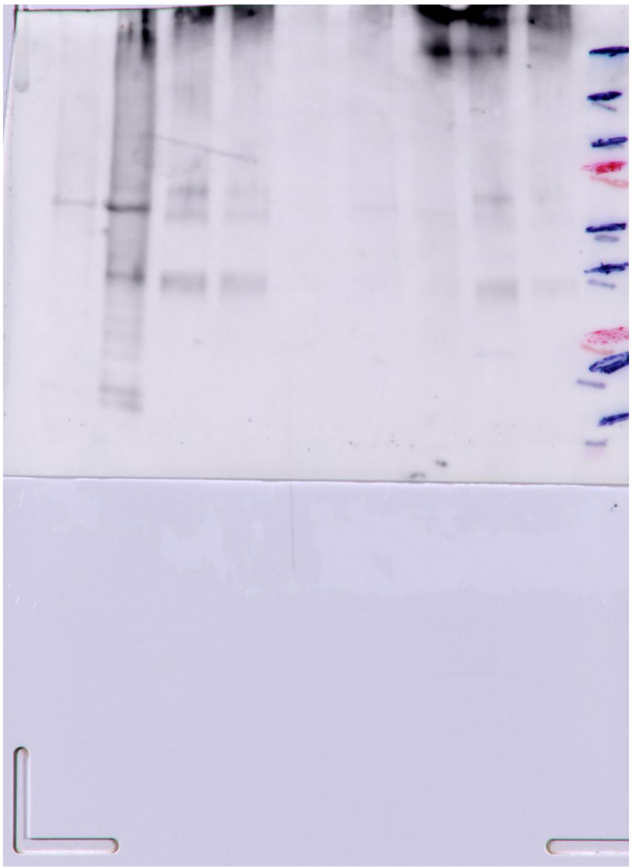

Figure S1. Western Blotting of the cytosolic (cyto) and membrane (mem) fractions purified from SH-SY5Y cells treated for 24 h with SF or OB* at 0.3 μM. αS species were then probed with conformation-insensitive anti-αS 211 antibodies.
